# Crystal structure and Hirshfeld surface analysis of a new benzodiazepine derivative: 4-di­chloro­methyl-2,3-di­hydro-1*H*-1,5-benzodiazepin-2-one

**DOI:** 10.1107/S205698901801681X

**Published:** 2019-01-01

**Authors:** Karim Chkirate, Sevgi Kansiz, Khalid Karrouchi, Joel T. Mague, Necmi Dege, El Mokhtar Essassi

**Affiliations:** a Laboratory of Heterocyclic Organic Chemistry URAC 21, Pole of Competence Pharmacochemistry, Av Ibn Battouta, BP 1014, Faculty of Sciences, Mohammed V University, Rabat, Morocco; b Ondokuz Mayıs University, Faculty of Arts and Sciences, Department of Physics, 55139, Kurupelit, Samsun, Turkey; c Physicochemical Service, Drugs Quality Control Laboratory, Division of Drugs and Pharmacy, Ministry of Health, 10100 Rabat, Morocco; d Department of Chemistry, Tulane University, New Orleans, LA 70118, USA

**Keywords:** crystal structure, benzodiazepin-2-one, phase-transfer catalysis, hydrogen bonding, C—H⋯π(ring) inter­action, Hirshfeld surface analysis

## Abstract

In the title compound, C_10_H_8_Cl_2_N_2_O, the seven-membered diazepine ring adopts a boat-shaped conformation. The mean planes of the two rings of the benzodiazepine unit are inclined to each other by 22.05 (6)°. In the crystal, mol­ecules are linked by pairs of N—H⋯O hydrogen bonds, forming inversion dimers with an *R* ^2^_2_(8) ring motif. The dimers are linked by C—H⋯π inter­actions, forming layers lying parallel to (10). The roles of the inter­molecular inter­actions in the crystal packing were clarified using Hirshfeld surface analysis; the most important contributions are from Cl⋯H/H⋯Cl (30.5%) and H⋯H (22.5%) inter­actions.

## Abstract 2

In the title compound, the seven-membered diazepine ring adopts a boat-shaped conformation. In the crystal, mol­ecules are linked by pairs of N—H⋯O hydrogen bonds, forming inversion dimers with an 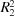
(8) ring motif.

## Chemical context   

Inter­est in benzodiazepines and their derivatives has concentrated on their pharmacological (Beaulieu, 2006 ▸; Tosti *et al.*, 2007 ▸) and chemical (Ahabchane & Essassi, 2000 ▸) properties. In addition, they are used as raw materials for the synthesis of substances with anti­bacterial (Essassi *et al.*, 1991 ▸) and anti­tumor (Lee *et al.*, 1978 ▸) activities. They are also used as secondary analgesics or as co-analgesics (Aveline *et al.*, 2001 ▸; Muster & Ben Slama, 2004 ▸). 1,5-Benzodiazepine derivatives have been shown to exhibit anti-inflammatory (Roma *et al.*, 1991 ▸), hypnotics (Kudo, 1982 ▸), anti-HIV-1 (Di Braccio *et al.*, 2001 ▸), anti­convulsant (De Sarro *et al.*, 1996 ▸), anti­microbial (Kumar & Joshi, 2007 ▸) and anti­tumor (Kamal *et al.*, 2008 ▸) activities. In a continuation of our work on the synthesis of 1,5-benzodiazepine derivatives (Chkirate *et al.*, 2018 ▸), we report herein on the synthesis and crystal structure of the title compound, 4-di­chloro­methyl-2,3-di­hydro-1*H*-1,5-benzodiaze­pin-2-one, together with the Hirshfeld surface analysis.
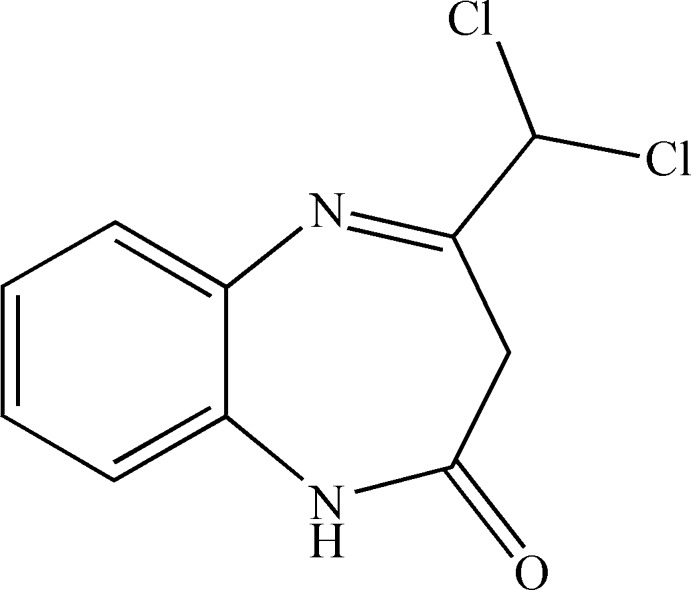


## Structural commentary   

**Figure 1:**
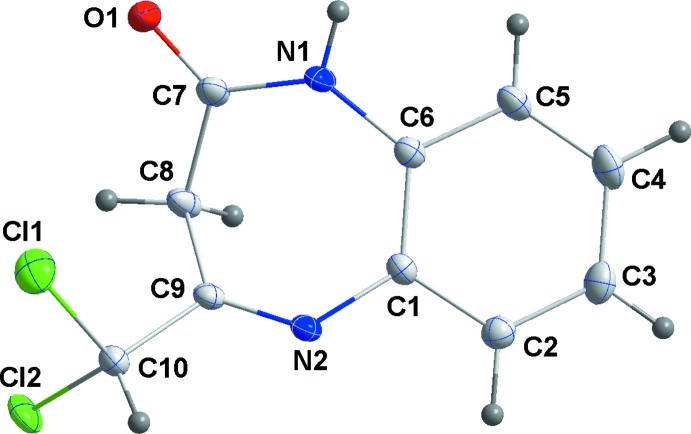
The mol­ecular structure of the title compound, with the atom labelling. Displacement ellipsoids are drawn at the 50% probability level.

The mol­ecular structure of the title compound is illustrated in Fig. 1 ▸. The seven-membered diazepine ring (C1/C6/N1/C7–C9/N2) adopts a boat-shaped conformation: puckering parameters are *Q*(2) = 0.7692 (14) Å, φ(2) = 21.25 (10)°, *Q*(3) = 0.2131 (14) Å, φ(3) = 131.2 (4)°, with a total puckering amplitude *Q* of 0.7982 (14) Å. The mean planes of the two rings are inclined to each other by 22.05 (6)°. The C9=N2 bond has a *Z* configuration and a bond length of 1.2737 (18) Å. The C1—N2 [1.4124 (17) Å] and C6—N1 [1.4068 (18) Å] bond lengths are typical for a 2,3-di­hydro-1*H*-1,5-benzodiazepin-2-one ring system and similar to those observed for the structure of a very similar compound, 4-methyl-2,3-di­hydro-1*H*-15-benzodiazepin-2-one monohydrate (Saber *et al.*, 2010 ▸); see also the *Database survey* section below.

## Supra­molecular features   

**Figure 2:**
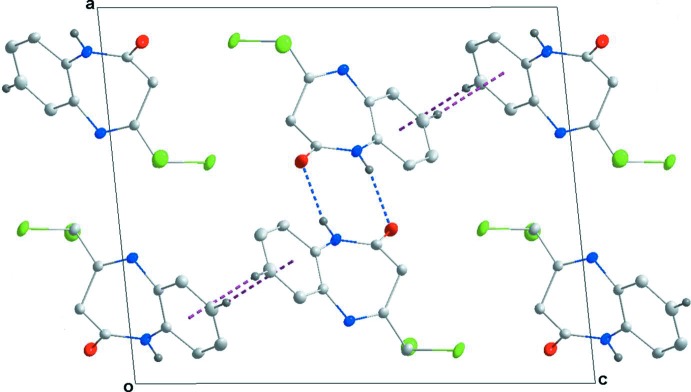
A partial view along the *b*-axis of the crystal packing of the title compound. The N—H⋯O hydrogen bonds are shown as blue dashed lines and the C—H⋯π(ring) inter­actions as purple dashed lines (see Table 1 ▸; H atoms not involved in these inter­actions have been omitted).

**Figure 3:**
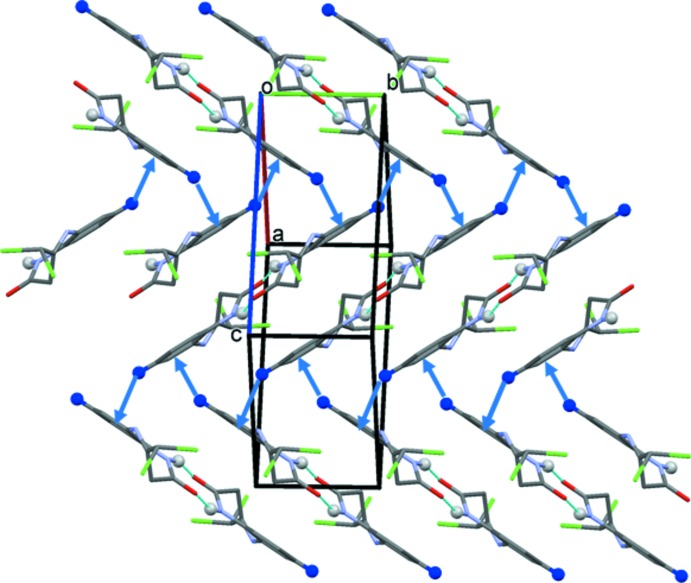
A view normal to (10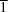) of the crystal packing of the title compound. The N—H⋯O hydrogen bonds are shown as dashed lines and the C—H⋯π inter­actions as blue arrows (see Table 1 ▸; H atoms not involved in these inter­actions have been omitted).

In the crystal, mol­ecules are linked by pairs of N—H⋯O hydrogen bonds, forming inversion dimers with an 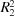
(8) ring motif (Table 1 ▸ and Fig. 2 ▸). The dimers are linked by C—H⋯π inter­actions, forming layers that lie parallel to the (10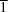) plane (Fig. 3 ▸ and Table 1 ▸). There are no other significant inter­molecular inter­actions present. The H⋯H or H⋯Cl inter­molecular distances all exceed the sum of their van der Waals radii.

## Database survey   

A search of the Cambridge Structural Database (CSD, version 5.39, update August 2018; Groom *et al.*, 2016 ▸) for the 2,3-di­hydro-1*H*-1,5-benzodiazepin-2-one skeleton yielded 12 hits (see supporting information). In all 12 compounds, the diazo­pine ring has a boat-shaped conformation, as does the title compound. The benzene ring and the mean plane of the diazepine ring are inclined to each other by dihedral angles ranging from *ca* 19.95 to 29.16°, compared to 22.05 (6)° in the title compound. The C=O bond lengths vary from *ca* 1.217–1.241 Å and the C=N bond lengths vary from *ca* 1.272–1.295 Å. In the title compound, the corresponding bond lengths are 1.2288 (18) and 1.2737 (18) Å, respectively. The C_aromatic_—N bond lengths in the diazepine ring range from *ca* 1.391 to 1.415 Å, compared to values of 1.4124 (17) and 1.4068 (18) Å for bonds C1—N2 and C6—N1, respectively, in the title compound. Hence, the various geometrical parameters mentioned above for the title compound are typical for 2,3-di­hydro-1*H*-1,5-benzodiazepin-2-ones. In the crystals of all but one compound, mol­ecules are linked by pairs of N—H⋯O hydrogen bonds, forming inversion dimers with an 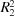
(8) ring motif. The same arrangement is observed in the crystal of the title compound.

## Hirshfeld surface analysis   

**Figure 4:**
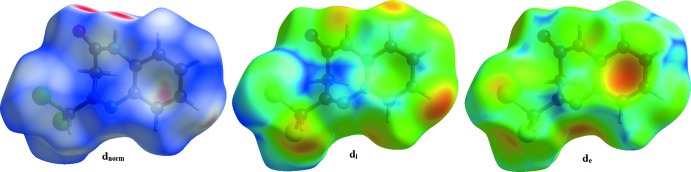
The Hirshfeld surfaces of the title compound mapped over *d*_norm_, *d*_i_ and *d*_e_.

The mol­ecular Hirshfeld surfaces were generated using a standard (high) surface resolution with the three-dimensional *d*_norm_ surfaces mapped over a fixed colour scale of −0.456 (red) to 1.092 (blue) Å using the *CrystalExplorer* program (Turner *et al.*, 2017 ▸). The red spots on the surface indicate the inter­molecular contacts involved in the hydrogen bonds. In Figs. 4 ▸ and 5 ▸, the red spots are attributed to the H⋯O close contacts.

**Figure 6:**
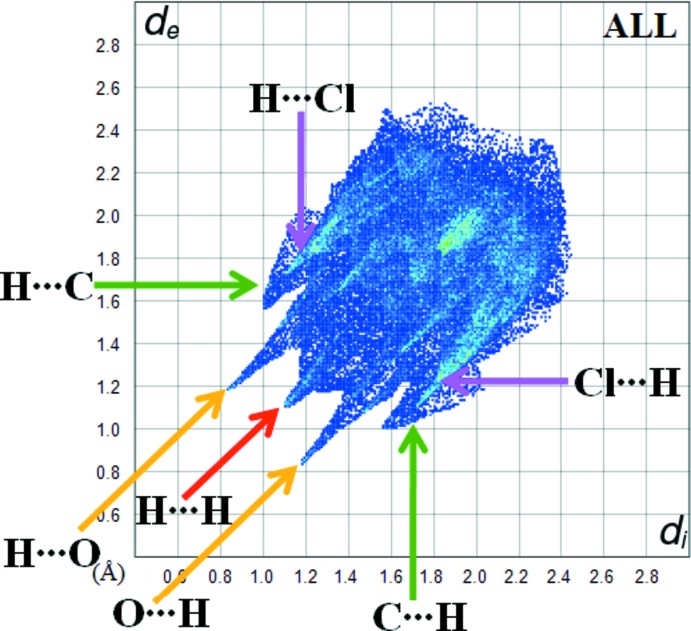
The overall fingerprint plot for the title compound.

**Figure 7:**
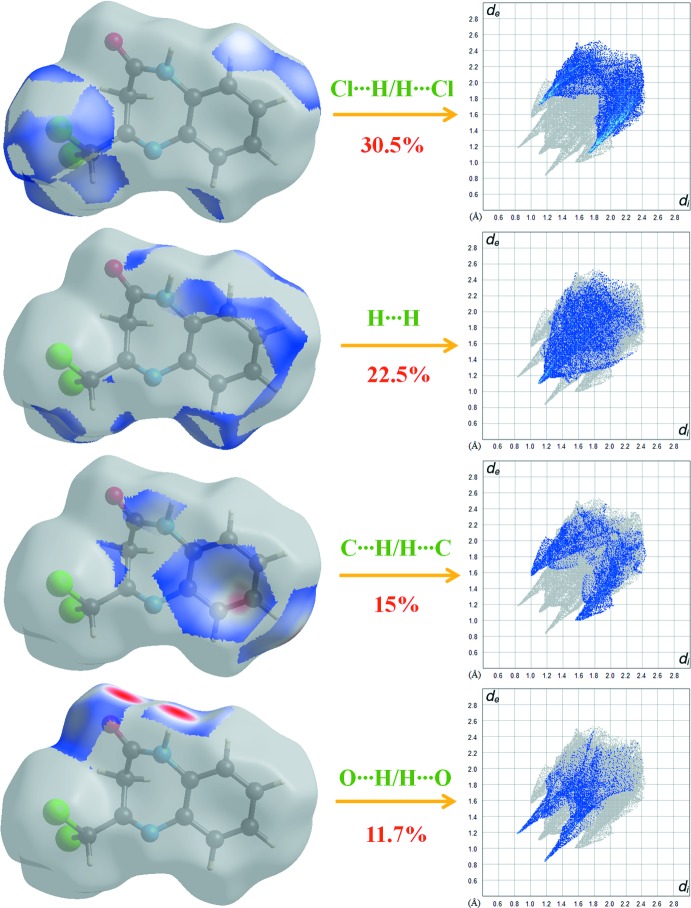
Two-dimensional fingerprint plots with a *d*_norm_ view of the Cl⋯H/H⋯Cl (30.5%), H⋯H (22.5%), C⋯H/H⋯C (15%) and O⋯H/H⋯O (5.5%) contacts in the title compound.

Fig. 6 ▸ shows the two-dimensional fingerprint plot for the sum of the contacts contributing to the Hirshfeld surface represented in normal mode. The graph shown in Fig. 7 ▸ represents the O⋯H/H⋯O contacts (30.4%) between the oxygen atoms inside the surface and the hydrogen atoms outside the surface at *d*_e_ + *d*_i_ = 2.5 Å and two symmetrical points at the top, bottom left and right. These data are characteristic of C—H⋯O hydrogen bonding.

The H⋯H graph in Fig. 7 ▸ shows the two-dimensional fingerprint of the (*d*_i_, *d*_e_) points associated with hydrogen atoms. It is characterized by an end point that points to the origin and corresponds to *d*_i_ = *d*_e_ = 1.08 Å, which indicates the presence of the H⋯H contacts in this study, which make a contribution of 54.3% to the crystal packing. The C⋯H/H⋯C graph in Fig. 7 ▸ shows the contacts between carbon atoms inside the Hirshfeld surface and hydrogen atoms outside and *vice versa* and has two symmetrical wings on the left and right sides (6.8%). Much weaker C⋯C (5.5%), O⋯N/N⋯O (2.4%), O⋯O (0.3%) and S⋯H/H⋯S (0.2%) contacts also occur.

**Figure 8:**
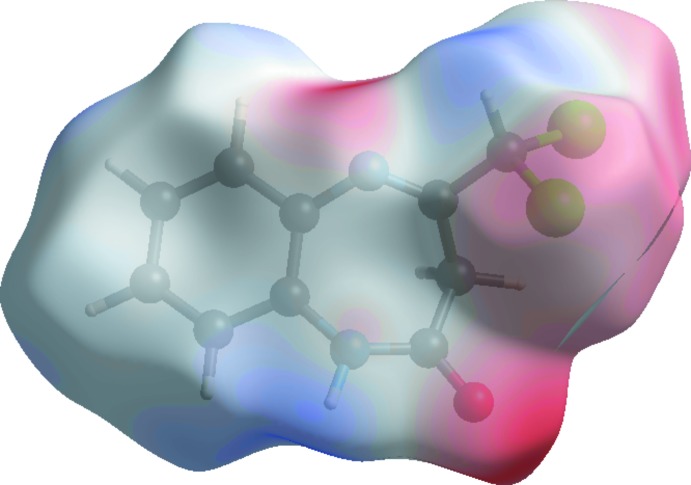
A view of the three-dimensional Hirshfeld surface plotted over electrostatic potential energy

A view of the three-dimensional Hirshfeld surface of the title compound plotted over electrostatic potential energy in the range −0.082 to 0.042 a.u. using the STO-3G basis set at the Hartree–Fock level of theory is shown in Fig. 8 ▸ where the N—H⋯O and C—H⋯π hydrogen-bond donors and acceptors are shown as blue and red areas around the atoms related with positive (hydrogen-bond donors) and negative (hydrogen-bond acceptors) electrostatic potentials, respectively.

## Synthesis and crystallization   

The title compound was synthesized by the reaction of di­chloro­methane with (*Z*)-4-(2-oxo­propyl­idene)-4,5-di­hydro-1*H*-benzo [*b*][1,5]-diazepine-2(3*H*)-one under phase-transfer catalysis (PTC) conditions using tetra-*n*-bromide butyl­ammonium (TBAB) as catalyst and potassium carbonate as base.

To a solution of 4-(2-oxo­propyl­idene)-4,5-di­hydro-*1H*-benzo-[*b*][1,5]diazepine-2(3*H*)-one (2.87 mmol) in di­chloro­methane (30 ml) as reagent and solvent, potassium carbonate (5.71 mmol) and a catalytic amount of tetra-*n*-butyl­ammonium bromide (0.37 mmol) were added. The mixture was stirred for 48 h. The solid material was removed by filtration and the solvent evaporated under vacuum. The residue was purified through silica gel column chromatography using hexa­ne/ethyl acetate (ratio 8:2). Slow evaporation at room temperature lead to the formation of colourless single crystals (yield 69%).

## Refinement   

Crystal data, data collection and structure refinement details are summarized in Table 2 ▸. All H atoms were located in a difference-Fourier map and freely refined.

## Supplementary Material

Crystal structure: contains datablock(s) global, I. DOI: 10.1107/S205698901801681X/xu5953sup1.cif

Structure factors: contains datablock(s) I. DOI: 10.1107/S205698901801681X/xu5953Isup2.hkl

Supporting information file. DOI: 10.1107/S205698901801681X/xu5953Isup4.cml

CSD search results. DOI: 10.1107/S205698901801681X/xu5953sup3.pdf

CCDC reference: 1881324

Additional supporting information:  crystallographic information; 3D view; checkCIF report

## Figures and Tables

**Figure 5:**
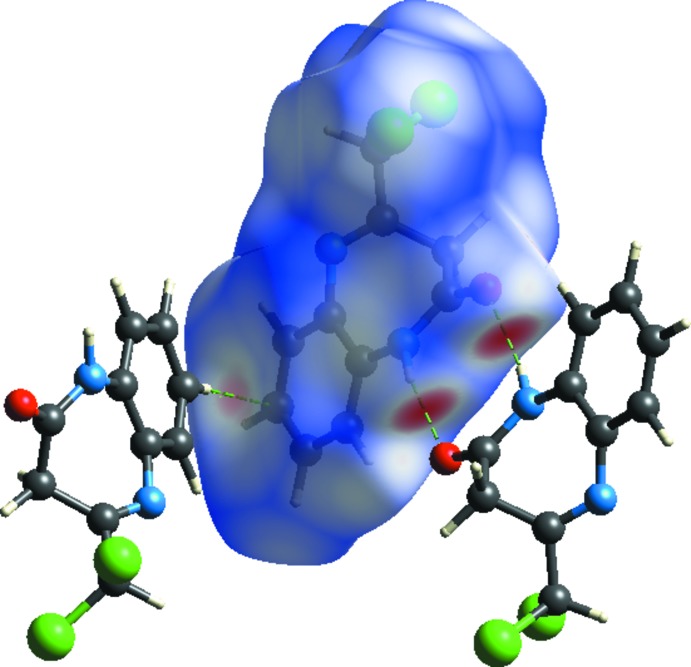
Hirshfeld surface mapped over *d*_norm_ to visualize the inter­molecular inter­actions.

**Table 1:** Hydrogen-bond geometry (Å, °) *Cg*1 is the centroid of the C1–C6 benzene ring.

*D*—H⋯*A*	*D*—H	H⋯*A*	*D*⋯*A*	*D*—H⋯*A*
N1—H1⋯O1^i^	0.86 (2)	2.15 (2)	2.977 (2)	160 (2)
C3—H3⋯*Cg*1^ii^	0.95 (2)	2.66 (2)	3.450 (2)	142 (1)

Symmetry codes: (i) 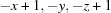; (ii) 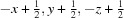.

**Table 2:** Experimental details

Crystal data	
Chemical formula	C_10_H_8_Cl_2_N_2_O
*M*_r_	243.08
Crystal system, space group	Monoclinic, *P*2_1_/*n*
Temperature (K)	150
*a*, *b*, *c* (Å)	12.1783 (6), 5.7217 (3), 14.8258 (7)
β (°)	95.740 (1)
*V* (Å^3^)	1027.89 (9)
*Z*	4
Radiation type	Cu *K*α
μ (mm^−1^)	5.46
Crystal size (mm)	0.33 × 0.24 × 0.14

Data collection	
Diffractometer	Bruker D8 VENTURE PHOTON 100 CMOS
Absorption correction	Multi-scan (*SADABS*; Krause *et al.*, 2015 ▸)
*T*_min_, *T*_max_	0.36, 0.51
No. of measured, independent and observed [*I* > 2σ(*I*)] reflections	7403, 2051, 2020
*R*_int_	0.028
(sin θ/λ)_max_ (Å^−1^)	0.625

Refinement	
*R*[*F* ^2^ > 2σ(*F* ^2^)], *wR*(*F* ^2^), *S*	0.028, 0.074, 1.05
No. of reflections	2051
No. of parameters	169
H-atom treatment	All H-atom parameters refined
Δρ_max_, Δρ_min_ (e Å^−3^)	0.32, −0.31

Computer programs: *APEX3* and *SAINT* (Bruker, 2016 ▸), *SHELXT* (Sheldrick, 2015*a* ▸), *SHELXL2018* (Sheldrick, 2015*b* ▸), *DIAMOND* (Brandenburg & Putz, 2012 ▸), *Mercury* (Macrae *et al.*, 2008 ▸) and *SHELXTL* (Sheldrick, 2008 ▸).

## supplementary crystallographic information

### Crystal data

**Table:**

C_10_H_8_Cl_2_N_2_O	*F*(000) = 496
*M**_r_* = 243.08	*D*_x_ = 1.571 Mg m^−^^3^
Monoclinic, *P*2_1_/*n*	Cu *K*α radiation, λ = 1.54178 Å
*a* = 12.1783 (6) Å	Cell parameters from 7262 reflections
*b* = 5.7217 (3) Å	θ = 3.0–74.6°
*c* = 14.8258 (7) Å	µ = 5.46 mm^−^^1^
β = 95.740 (1)°	*T* = 150 K
*V* = 1027.89 (9) Å^3^	Block, colourless
*Z* = 4	0.33 × 0.24 × 0.14 mm

### Data collection

**Table:**

Bruker D8 VENTURE PHOTON 100 CMOS diffractometer	2051 independent reflections
Radiation source: INCOATEC IµS micro-focus source	2020 reflections with *I* > 2σ(*I*)
Mirror monochromator	*R*_int_ = 0.028
Detector resolution: 10.4167 pixels mm^-1^	θ_max_ = 74.6°, θ_min_ = 4.5°
ω scans	*h* = −15→15
Absorption correction: multi-scan (*SADABS*; Krause *et al.*, 2015)	*k* = −6→7
*T*_min_ = 0.36, *T*_max_ = 0.51	*l* = −18→17
7403 measured reflections	

### Refinement

**Table:**

Refinement on *F*^2^	Secondary atom site location: difference Fourier map
Least-squares matrix: full	Hydrogen site location: difference Fourier map
*R*[*F*^2^ > 2σ(*F*^2^)] = 0.028	All H-atom parameters refined
*wR*(*F*^2^) = 0.074	*w* = 1/[σ^2^(*F*_o_^2^) + (0.0392*P*)^2^ + 0.599*P*] where *P* = (*F*_o_^2^ + 2*F*_c_^2^)/3
*S* = 1.05	(Δ/σ)_max_ = 0.001
2051 reflections	Δρ_max_ = 0.32 e Å^−^^3^
169 parameters	Δρ_min_ = −0.31 e Å^−^^3^
0 restraints	Extinction correction: *SHELXL2018* (Sheldrick, 2015*b*), Fc^*^=kFc[1+0.001xFc^2^λ^3^/sin(2θ)]^-1/4^
Primary atom site location: structure-invariant direct methods	Extinction coefficient: 0.0137 (7)

### Special details

**Table:**

Geometry. All esds (except the esd in the dihedral angle between two l.s. planes) are estimated using the full covariance matrix. The cell esds are taken into account individually in the estimation of esds in distances, angles and torsion angles; correlations between esds in cell parameters are only used when they are defined by crystal symmetry. An approximate (isotropic) treatment of cell esds is used for estimating esds involving l.s. planes.
Refinement. Refinement of F^2^ against ALL reflections. The weighted R-factor wR and goodness of fit S are based on F^2^, conventional R-factors R are based on F, with F set to zero for negative F^2^. The threshold expression of F^2^ > 2sigma(F^2^) is used only for calculating R-factors(gt) etc. and is not relevant to the choice of reflections for refinement. R-factors based on F^2^ are statistically about twice as large as those based on F, and R- factors based on ALL data will be even larger.

### Fractional atomic coordinates and isotropic or equivalent isotropic displacement parameters (Å^2^)

**Table:**

	*x*	*y*	*z*	*U*_iso_*/*U*_eq_	
Cl1	0.09759 (3)	−0.04560 (6)	0.60350 (3)	0.02813 (14)	
Cl2	0.08781 (3)	0.37781 (6)	0.71005 (2)	0.02306 (14)	
O1	0.40917 (8)	−0.0195 (2)	0.59019 (7)	0.0239 (2)	
N1	0.38241 (9)	0.2146 (2)	0.46776 (8)	0.0168 (3)	
H1	0.4316 (17)	0.132 (4)	0.4445 (14)	0.031 (5)*	
N2	0.16589 (9)	0.4612 (2)	0.47917 (8)	0.0146 (2)	
C1	0.25109 (10)	0.5411 (2)	0.42893 (9)	0.0140 (3)	
C2	0.22893 (11)	0.7435 (3)	0.37666 (9)	0.0181 (3)	
H2	0.1585 (16)	0.826 (4)	0.3825 (13)	0.030 (5)*	
C3	0.30118 (12)	0.8234 (3)	0.31737 (10)	0.0210 (3)	
H3	0.2849 (15)	0.963 (3)	0.2844 (13)	0.021 (4)*	
C4	0.39584 (12)	0.6952 (3)	0.30557 (10)	0.0220 (3)	
H4	0.4448 (16)	0.745 (3)	0.2612 (13)	0.025 (4)*	
C5	0.41957 (11)	0.4960 (3)	0.35629 (9)	0.0187 (3)	
H5	0.4819 (17)	0.403 (3)	0.3483 (13)	0.026 (5)*	
C6	0.35036 (11)	0.4207 (2)	0.42047 (9)	0.0144 (3)	
C7	0.36854 (10)	0.1586 (3)	0.55461 (9)	0.0168 (3)	
C8	0.30141 (11)	0.3298 (3)	0.60421 (9)	0.0182 (3)	
H8A	0.3361 (17)	0.485 (4)	0.6036 (14)	0.032 (5)*	
H8B	0.2984 (15)	0.268 (3)	0.6639 (14)	0.024 (4)*	
C9	0.18779 (10)	0.3589 (2)	0.55513 (9)	0.0139 (3)	
C10	0.09010 (11)	0.2661 (2)	0.59849 (9)	0.0166 (3)	
H10	0.0224 (15)	0.308 (3)	0.5653 (12)	0.016 (4)*	

### Atomic displacement parameters (Å^2^)

**Table:**

	*U*^11^	*U*^22^	*U*^33^	*U*^12^	*U*^13^	*U*^23^
Cl1	0.0370 (2)	0.0167 (2)	0.0315 (2)	−0.00336 (14)	0.00773 (16)	−0.00190 (14)
Cl2	0.0278 (2)	0.0245 (2)	0.0190 (2)	−0.00043 (13)	0.01315 (14)	−0.00376 (13)
O1	0.0217 (5)	0.0304 (6)	0.0204 (5)	0.0108 (4)	0.0055 (4)	0.0079 (4)
N1	0.0161 (5)	0.0205 (6)	0.0145 (6)	0.0065 (5)	0.0052 (4)	0.0004 (5)
N2	0.0135 (5)	0.0151 (6)	0.0156 (5)	0.0019 (4)	0.0042 (4)	−0.0011 (4)
C1	0.0135 (6)	0.0159 (7)	0.0129 (6)	−0.0013 (5)	0.0027 (5)	−0.0012 (5)
C2	0.0187 (6)	0.0172 (7)	0.0183 (7)	0.0011 (5)	0.0019 (5)	−0.0001 (5)
C3	0.0257 (7)	0.0172 (7)	0.0201 (7)	−0.0044 (6)	0.0015 (5)	0.0029 (6)
C4	0.0198 (7)	0.0273 (8)	0.0196 (7)	−0.0087 (6)	0.0051 (5)	0.0013 (6)
C5	0.0136 (6)	0.0252 (7)	0.0180 (7)	−0.0019 (5)	0.0043 (5)	−0.0010 (6)
C6	0.0134 (6)	0.0171 (6)	0.0129 (6)	−0.0005 (5)	0.0013 (5)	−0.0019 (5)
C7	0.0119 (6)	0.0233 (7)	0.0151 (6)	0.0025 (5)	0.0012 (5)	0.0005 (5)
C8	0.0142 (6)	0.0281 (8)	0.0125 (6)	0.0044 (5)	0.0019 (5)	−0.0015 (6)
C9	0.0132 (6)	0.0153 (6)	0.0136 (6)	0.0024 (5)	0.0034 (5)	−0.0025 (5)
C10	0.0166 (6)	0.0177 (7)	0.0162 (6)	0.0014 (5)	0.0055 (5)	0.0010 (5)

### Geometric parameters (Å, º)

**Table:**

Cl1—C10	1.7868 (15)	C3—C4	1.392 (2)
Cl2—C10	1.7761 (14)	C3—H3	0.95 (2)
O1—C7	1.2288 (18)	C4—C5	1.380 (2)
N1—C7	1.3539 (18)	C4—H4	0.97 (2)
N1—C6	1.4068 (18)	C5—C6	1.4006 (19)
N1—H1	0.86 (2)	C5—H5	0.94 (2)
N2—C9	1.2737 (18)	C7—C8	1.5130 (19)
N2—C1	1.4124 (17)	C8—C9	1.5068 (18)
C1—C2	1.405 (2)	C8—H8A	0.99 (2)
C1—C6	1.4080 (18)	C8—H8B	0.96 (2)
C2—C3	1.382 (2)	C9—C10	1.5045 (18)
C2—H2	0.99 (2)	C10—H10	0.949 (18)
			
C7—N1—C6	128.15 (12)	C5—C6—C1	119.36 (13)
C7—N1—H1	114.2 (14)	N1—C6—C1	124.31 (12)
C6—N1—H1	115.4 (14)	O1—C7—N1	121.44 (13)
C9—N2—C1	121.01 (11)	O1—C7—C8	122.85 (13)
C2—C1—C6	118.27 (12)	N1—C7—C8	115.71 (12)
C2—C1—N2	116.55 (11)	C9—C8—C7	110.57 (11)
C6—C1—N2	124.84 (12)	C9—C8—H8A	105.7 (12)
C3—C2—C1	121.60 (13)	C7—C8—H8A	109.1 (12)
C3—C2—H2	120.6 (12)	C9—C8—H8B	111.6 (11)
C1—C2—H2	117.7 (12)	C7—C8—H8B	106.6 (12)
C2—C3—C4	119.59 (14)	H8A—C8—H8B	113.3 (17)
C2—C3—H3	119.6 (11)	N2—C9—C10	115.81 (11)
C4—C3—H3	120.8 (11)	N2—C9—C8	125.35 (12)
C5—C4—C3	119.84 (13)	C10—C9—C8	118.82 (12)
C5—C4—H4	120.1 (12)	C9—C10—Cl2	110.96 (10)
C3—C4—H4	120.1 (12)	C9—C10—Cl1	109.31 (9)
C4—C5—C6	121.12 (13)	Cl2—C10—Cl1	109.03 (7)
C4—C5—H5	121.6 (12)	C9—C10—H10	111.8 (11)
C6—C5—H5	117.3 (12)	Cl2—C10—H10	107.3 (10)
C5—C6—N1	116.14 (12)	Cl1—C10—H10	108.4 (11)
			
C9—N2—C1—C2	−147.75 (13)	N2—C1—C6—N1	−6.1 (2)
C9—N2—C1—C6	39.04 (19)	C6—N1—C7—O1	−173.53 (13)
C6—C1—C2—C3	0.7 (2)	C6—N1—C7—C8	5.9 (2)
N2—C1—C2—C3	−172.94 (13)	O1—C7—C8—C9	−122.19 (15)
C1—C2—C3—C4	3.1 (2)	N1—C7—C8—C9	58.41 (16)
C2—C3—C4—C5	−3.2 (2)	C1—N2—C9—C10	−175.53 (11)
C3—C4—C5—C6	−0.6 (2)	C1—N2—C9—C8	5.9 (2)
C4—C5—C6—N1	179.62 (13)	C7—C8—C9—N2	−70.12 (18)
C4—C5—C6—C1	4.4 (2)	C7—C8—C9—C10	111.35 (14)
C7—N1—C6—C5	146.34 (14)	N2—C9—C10—Cl2	−124.87 (11)
C7—N1—C6—C1	−38.7 (2)	C8—C9—C10—Cl2	53.79 (15)
C2—C1—C6—C5	−4.42 (19)	N2—C9—C10—Cl1	114.86 (12)
N2—C1—C6—C5	168.68 (12)	C8—C9—C10—Cl1	−66.48 (14)
C2—C1—C6—N1	−179.21 (12)		

### Hydrogen-bond geometry (Å, º)

Cg1 is the centroid of the C1–C6 benzene ring.

**Table:**

*D*—H···*A*	*D*—H	H···*A*	*D*···*A*	*D*—H···*A*
N1—H1···O1^i^	0.86 (2)	2.15 (2)	2.977 (2)	160 (2)
C3—H3···*Cg*1^ii^	0.95 (2)	2.66 (2)	3.450 (2)	142 (1)

Symmetry codes: (i) −*x*+1, −*y*, −*z*+1; (ii) −*x*+1/2, *y*+1/2, −*z*+1/2.
