# Supplementary material for: Crystal structure and Hirshfeld surface analysis of a new benzodiazepine derivative: 4-di­chloro­methyl-2,3-di­hydro-1H-1,5-benzodiazepin-2-one
Source: Acta Crystallogr E Crystallogr Commun. 2019 Jan 1;75(Pt 1):33–7. doi: 10.1107/S205698901801681X (PMC6323885; doi:10.1107/S205698901801681X)

Search Overview

Search:	search3
Date/Time done:	Wed Nov 28 19:16:15 2018
Database(s):	CSD version 5.39 updates (Nov 2017)<br>CSD version 5.39 (November 2017)<br>CSD version 5.39 (November 2017)<br>CSD version 5.39 updates (Feb 2018)<br>CSD version 5.39 updates (May 2018)<br>CSD version 5.39 updates (Aug 2018)
Restriction Info:	No refcode restrictions applied
Filters:	None
Percentage Completed:	100%
Number of Hits:	12

Single query used. Search found structures that:

match

Query 1

Query 1

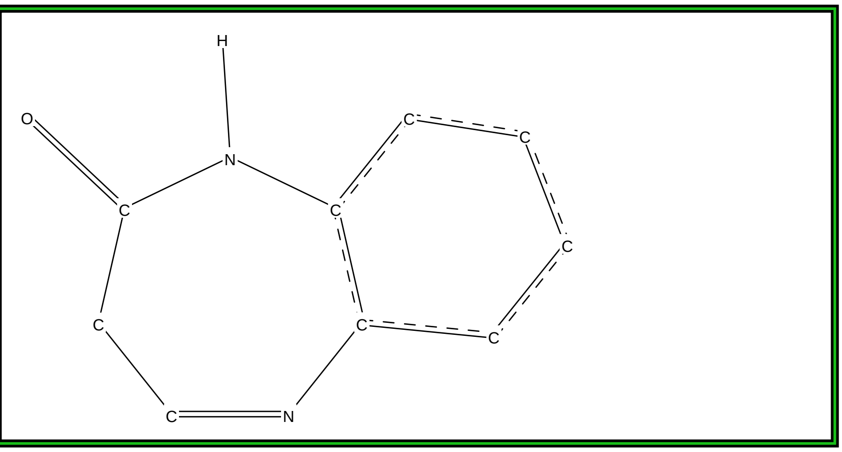

Search: search3 (Wed Nov 28 19:16:15 2018): Hits 1-4

EFARUA

Reference: M.Akkurt, A.R.Kennedy, S.H.H.Younes, S.K.Mohamed, A.A.Abdelhamid (2012) *Acta Crystallogr.,Sect.E:Struct.Rep.Online* ,**68,** o3356

Formula: $C_{15}\,H_{12}\,N_2\,O_1$

Compound Name: 4-Phenyl-1H-1,5-benzodiazepin-2(3H)-one

Space Group:	P-1	***Cell:***	***a*** 4.689(0)	***b*** 10.835(1)	***c*** 11.754(1)
Space Group No.:	2	***(Å,°)***	α 77.72(1)	β 83.81(0)	γ 82.11(1)

R-Factor (%): 5.34 ***Temperature(K):*** 123 ***Density(g/cm***^{3}***):*** 1.362

Parameters

Fragment 1
ANG1 (A) 20.251
DIST1 (D) 1.237
DIST2 (D) 1.285
DIST3 (D) 1.412
DIST4 (D) 1.402

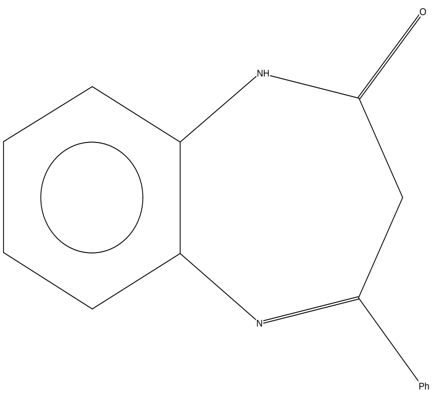

INIZUC

Reference: H.Mtiraoui, R.Gharbi, M.Msaddek, Y.Bretonniere, C.Andraud, C.Sabot, P.-Y.Renard (2016) *J.Org.Chem.* ,**81,**4720

Formula: $C_{15}\,H_{12}\,N_2\,O_2$

Compound Name: 4-(2-hydroxyphenyl)-1,3-dihydro-2H-1,5-benzodiazepin-2-one

Space Group:	P21/n	***Cell:***	***a*** 4.692(0)	***b*** 12.148(2)	***c*** 21.030(4)
Space Group No.:	14	***(Å,°)***	α 90.00	β 95.01(0)	γ 90.00

R-Factor (%): 4.20 ***Temperature(K):*** 293 ***Density(g/cm***^{3}***):*** 1.403

Parameters

Fragment 1
ANG1 (A) 19.948
DIST1 (D) 1.231
DIST2 (D) 1.290
DIST3 (D) 1.405
DIST4 (D) 1.402

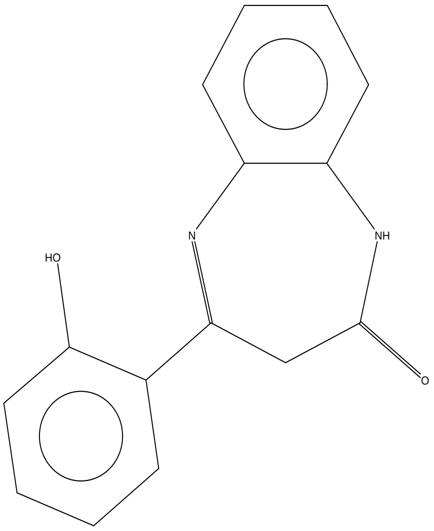

JARBOW

Reference: O.Martin, M.Perez-Torralba, M.Angeles Garcia, R.M.Claramunt, M.Carmen Torralba, M.Rosario Torres, I.Alkorta, J.Elguero (2016) *Chem. Sel.* ,**1,**861

Formula: $C_{15}\,H_8\,F_4\,N_2\,O_1$

Compound Name: 6,7,8,9-tetrafluoro-4-phenyl-1,3-dihydro-2H-1,5-benzodiazepin-2-one

Space Group:	P21/c	***Cell:***	***a*** 13.035(3)	***b*** 6.981(1)	***c*** 15.450(4)
Space Group No.:	14	***(Å,°)***	α 90.00	β 110.70(0)	γ 90.00

R-Factor (%): 4.34 ***Temperature(K):*** 296 ***Density(g/cm***^{3}***):*** 1.557

Parameters

Fragment 1
ANG1 (A) 22.295
DIST1 (D) 1.225
DIST2 (D) 1.286
DIST3 (D) 1.400
DIST4 (D) 1.397

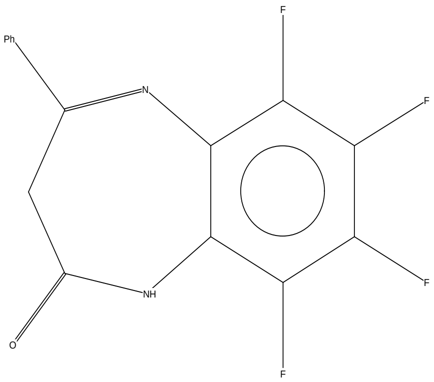

JARBUC

Reference: O.Martin, M.Perez-Torralba, M.Angeles Garcia, R.M.Claramunt, M.Carmen Torralba, M.Rosario Torres, I.Alkorta, J.Elguero (2016) *Chem. Sel.* ,**1,**861

Formula: $C_{15}\,H_7\,F_5\,N_2\,O_1$

Compound Name: 6,7,8,9-tetrafluoro-4-(2-fluorophenyl)-1,3-dihydro-2H-1,5-benzodiazepin-2-one

Space Group:	P21/n	***Cell:***	***a*** 6.502	***b*** 25.870	***c*** 7.913
Space Group No.:	14	***(Å,°)***	α 90.00	β 106.87	γ 90.00

R-Factor (%): 4.07 ***Temperature(K):*** 293 ***Density(g/cm***^{3}***):*** 1.701

Parameters

Fragment 1
ANG1 (A) 23.528
DIST1 (D) 1.217
DIST2 (D) 1.282
DIST3 (D) 1.403
DIST4 (D) 1.403

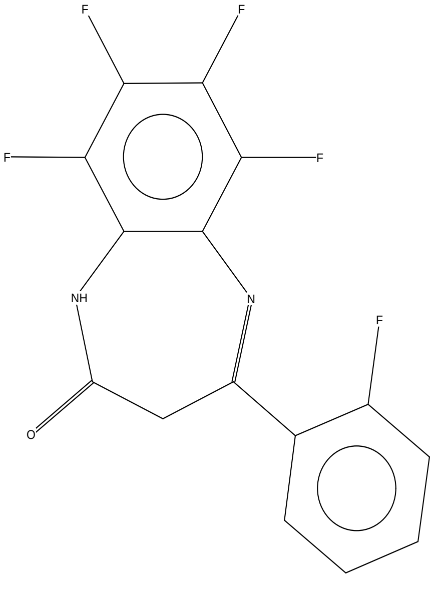

Search: search3 (Wed Nov 28 19:16:15 2018): Hits 5-8

JARCAJ

Reference: O.Martin, M.Perez-Torralba, M.Angeles Garcia, R.M.Claramunt, M.Carmen Torralba, M.Rosario Torres, I.Alkorta, J.Elguero (2016) *Chem. Sel.* ,**1,**861

Formula: $C_{15}\,H_7\,Cl_1\,F_4\,N_2\,O_1$

Compound Name: 4-(2-chlorophenyl)-6,7,8,9-tetrafluoro-1,3-dihydro-2H-1,5-benzodiazepin-2-one

Space Group:	P-1	***Cell:***	***a***	7.654(2)	***b*** 7.845(2)	***c*** 12.462(3)
Space Group No.:	2	***(Å,°)***	α	93.29(0)	β 106.39(0)	γ 108.61(0)

R-Factor (%): 4.42 ***Temperature(K):*** 296 ***Density(g/cm^3):*** 1.695

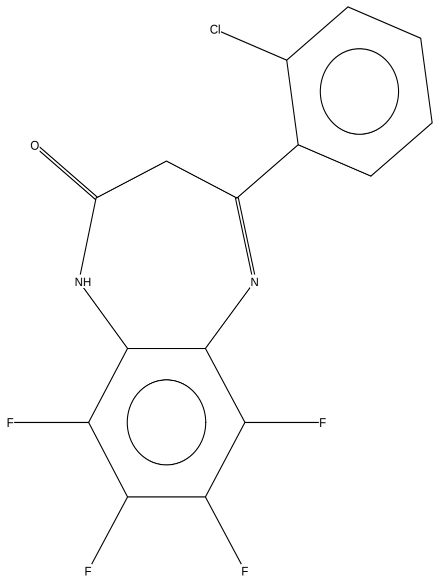

Parameters

Fragment 1

**ANG1 (A)**	22.785
**DIST1 (D)**	1.219
**DIST2 (D)**	1.279
**DIST3 (D)**	1.407
**DIST4 (D)**	1.391

MOMBAT

Reference: M.Perez-Torralba, R.M.Claramunt, M.Angeles Garcia, C.Lopez, M.Carmen Torralba, M.Rosario Torres, I.Alkorta, J.Elguero (2013) *Beilstein J.Org.Chem.* ,**9,**2156

Formula: $C_{10}\,H_6\,F_4\,N_2\,O_1$

Compound Name: 6,7,8,9-Tetrafluoro-4-methyl-1,3-dihydro-2H-1,5-benzodiazepin-2-one

Space Group:	P21/c	***Cell:***	***a***	5.282(0)	***b*** 18.255(1)	***c*** 10.210(0)
Space Group No.:	14	***(Å,°)***	α	90.00	β 100.93(1)	γ 90.00

R-Factor (%): 3.86 ***Temperature(K):*** 293 ***Density(g/cm^3):*** 1.691

Parameters

Fragment 1

**ANG1 (A)**	21.860
**DIST1 (D)**	1.219
**DIST2 (D)**	1.273
**DIST3 (D)**	1.401
**DIST4 (D)**	1.405

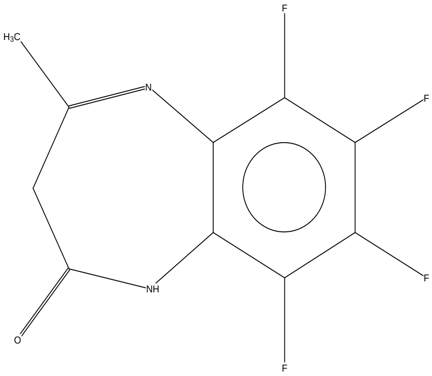

TALSEH

Reference: A.Essaghouani, M.Boulhaoua, S.Lahmidi, M.M.Mohamed Abdelahi, E.M.Essassi, J.T.Mague (2017) *IUCrData* ,**2,** x170389

Formula: $C_{15}\,H_{10}\,Br_2\,N_2\,O_1$

Compound Name: (3S)-3,8-dibromo-4-phenyl-2,3-dihydro-1H-1,5-benzodiazapine-2-one

Space Group:	P-1	***Cell:***	***a***	7.893(0)	***b*** 9.930(1)	***c*** 10.241(1)
Space Group No.:	2	***(Å,°)***	α	101.75(0)	β 105.97(0)	γ 109.48(0)

R-Factor (%): 2.82 ***Temperature(K):*** 100 ***Density(g/cm^3):*** 1.903

Parameters

Fragment 1

**ANG1 (A)**	24.370
**DIST1 (D)**	1.235
**DIST2 (D)**	1.284
**DIST3 (D)**	1.411
**DIST4 (D)**	1.406

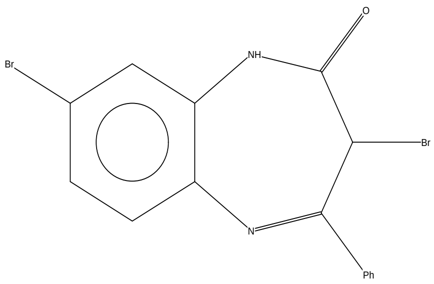

TUPSAZ

Reference: A.Saber, H.Zouihri, E.M.Essassi, S.W.Ng (2010) *Acta Crystallogr.,Sect.E:Struct.Rep.Online* ,**66,**o1408

Formula: $C_{10}\,H_{10}\,N_2\,O_1,H_2\,O_1$

Compound Name: 4-Methyl-2,3-dihydro-1H-1,5-benzodiazepin-2-one monohydrate

Space Group:	P-1	***Cell:***	***a***	4.901(0)	***b*** 7.315(0)	***c*** 13.569(0)
Space Group No.:	2	***(Å,°)***	α	85.38(0)	β 83.96(0)	γ 83.81(0)

R-Factor (%): 3.81 ***Temperature(K):*** 100 ***Density(g/cm^3):*** 1.331

Parameters

Fragment 1

**ANG1 (A)**	22.319
**DIST1 (D)**	1.235
**DIST2 (D)**	1.278
**DIST3 (D)**	1.409
**DIST4 (D)**	1.411

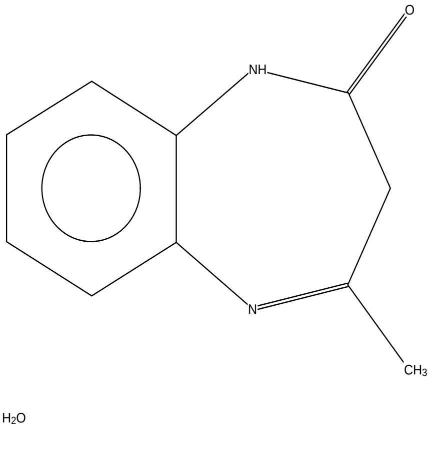

Search: search3 (Wed Nov 28 19:16:15 2018): Hits 9-12

XOTQAA

Reference: S.CHNITI (2014)
CSD Communication(Private Communication) ,

Formula: $C_{20}\,H_{21}\,N_3\,O_2$

Compound Name: 3-((butylamino)methylene)-4-(2-hydroxyphenyl)-1,3-dihydro-2H-1,5-benzodiazepin-2-one

Space Group:	C2/c	***Cell:***	***a***	26.091(1)	***b***	9.078(0)	***c***	15.143(0)
Space Group No.:	15	***(Å,°)***	α	90.00	β	97.26(0)	γ	90.00

R-Factor (%): 6.32 ***Temperature(K):*** 293 ***Density(g/cm3):*** 1.252

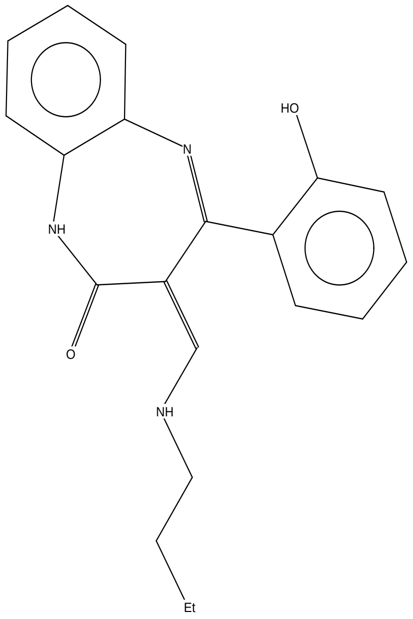

Parameters

Fragment 1

**ANG1 (A)**	22.278
**DIST1 (D)**	1.241
**DIST2 (D)**	1.295
**DIST3 (D)**	1.412
**DIST4 (D)**	1.400

ZISQOJ

Reference: M.Loughzail, A.Baouid, J.A.Fernandes, M.Driss, E.H.Soumhi (2014) *Acta Crystallogr.,Sect.E:Struct.Rep.Online* ,**70,**o126

Formula: $C_{18}\,H_{17}\,N_3\,O_1$

Compound Name: (E)-3-((Dimethylamino)methylene)-4-phenyl-1,3-dihydro-2H-1,5-benzodiazepin-2-one

Space Group:	P21/n	***Cell:***	***a***	11.281(2)	***b***	14.005(4)	***c***	20.124(3)
Space Group No.:	14	***(Å,°)***	α	90.00	β	95.97(1)	γ	90.00

R-Factor (%): 5.15 ***Temperature(K):*** 300 ***Density(g/cm3):*** 1.224

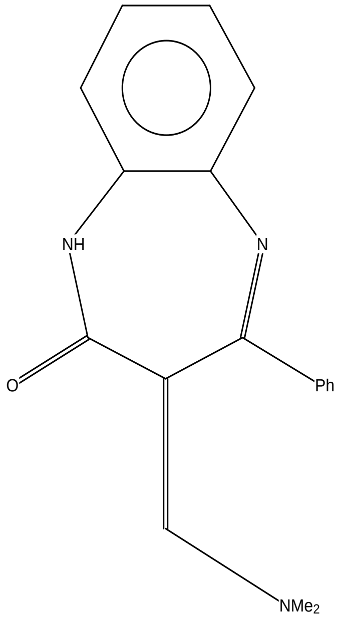

Parameters

Fragment 1

**ANG1 (A)**	25.170
**DIST1 (D)**	1.234
**DIST2 (D)**	1.285
**DIST3 (D)**	1.404
**DIST4 (D)**	1.405

Fragment 2

**ANG1 (A)**	29.161
**DIST1 (D)**	1.229
**DIST2 (D)**	1.285
**DIST3 (D)**	1.410
**DIST4 (D)**	1.404

ZUFQAU

Reference: A.Nsira, R.Gharbi, M.Msaddek (2014)
Comptes Rendus Chimie ,**17,**1242

Formula: $C_{18}\,H_{16}\,N_2\,O_3,C_5\,H_6\,N_2$

Compound Name: 6-ethoxy-6a,8-dihydrochromeno[4,3-b][1,5]benzodiazepin-7(6H)-one pyridin-2-amine

Space Group:	P-1	***Cell:***	***a***	6.849(5)	***b***	10.387(5)	***c***	15.298(5)
Space Group No.:	2	***(Å,°)***	α	106.25(0)	β	91.10(0)	γ	97.94(0)

R-Factor (%): 4.20 ***Temperature(K):*** 150 ***Density(g/cm3):*** 1.294

Parameters

Fragment 1

**ANG1 (A)**	25.488
**DIST1 (D)**	1.232
**DIST2 (D)**	1.282
**DIST3 (D)**	1.415
**DIST4 (D)**	1.414

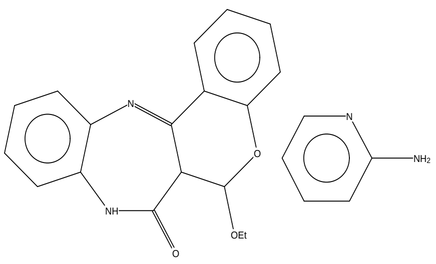

GESFOC

Reference: H.Mtiraoui, A.Nsira, M.Msaddek, P.-Y.Renard, C.Sabot (2017) *Comptes Rendus Chimie* ,**20,**747

Formula: $C_{17}\,H_{14}\,N_2\,O_3$

Compound Name: 2-(2-oxo-2,3-dihydro-1H-1,5-benzodiazepin-4-yl)phenyl acetate

Space Group:	P21/c	***Cell:***	***a***	6.240(1)	***b***	13.015(2)	***c***	17.705(3)
Space Group No.:	14	***(Å,°)***	α	90.00	β	97.00(0)	γ	90.00

R-Factor (%): 5.49 ***Temperature(K):*** 293 ***Density(g/cm3):*** 1.370

Parameters

Fragment 1

**ANG1 (A)**	25.022
**DIST1 (D)**	1.235
**DIST2 (D)**	1.278
**DIST3 (D)**	1.415
**DIST4 (D)**	1.406

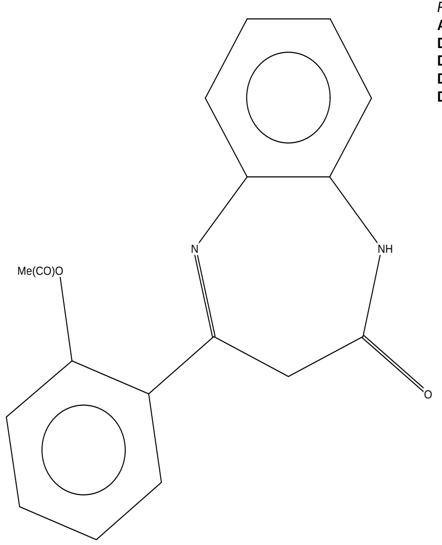

Supplement: Supplementary file 4 [file e-75-00033-sup3.pdf]
